# Motion-Informed, Patient-Specific Femoral Localization for MPFL Reconstruction Using 4D-CT and Constrained Optimization

**DOI:** 10.3390/diagnostics16040508

**Published:** 2026-02-07

**Authors:** Jiaying Wei, Xinhao Zhang, Jia Li, Weigen Ye, Runxing Kang, Dehua Wang, Weilin Wu, Mao Yuan, Yinsong Sun, Hong Cheng, Wei Huang, Ke Li, Chaobin Zou, Chen Zhao

**Affiliations:** 1 Department of Orthopedics, The First Affiliated Hospital of Chongqing Medical University, Chongqing 400016, China; weijy0929@163.com (J.W.); kangrx_double@163.com (R.K.); 17853591682@163.com (D.W.); sunyinsongcqmu@163.com (Y.S.); li.ke-ortho@hotmail.com (K.L.); 2 Chongqing Municipal Health Commission Key Laboratory of Musculoskeletal Regeneration and Translational Medicine, Chongqing 400016, China; 3 Orthopaedic Research Laboratory of Chongqing Medical University, Chongqing 400016, China; 4 School of Automation Engineering, University of Electronic Science and Technology of China, Chengdu 611731, China; xhzhang_uestc@163.com (X.Z.); 2022190501013@std.uestc.edu.cn (W.Y.); wuweilin31@gmail.com (W.W.); hcheng@uestc.edu.cn (H.C.); chaobinzou@uestc.edu.cn (C.Z.); 5 Department of Radiology, The First Affiliated Hospital of Chongqing Medical University, Chongqing 400016, China; 2h3864@hospital.cqmu.edu.cn (J.L.); miayuan113@163.com (M.Y.)

**Keywords:** four-dimensional computed tomography, patellofemoral kinematics, motion-informed analysis, patient-specific femoral localization, medial patellofemoral ligament

## Abstract

**Background:** Accurate femoral localization is a critical factor influencing graft length-change behavior in medial patellofemoral ligament reconstruction (MPFLR). However, the commonly used Schöttle point is derived from static radiographs and does not account for subject-specific patellofemoral kinematics during active knee motion. In this study, we integrated four-dimensional computed tomography (4D-CT) with constrained optimization to establish a motion-informed, patient-specific femoral localization framework. **Methods:** A total of 1382 4D-CT knee datasets were screened, and 58 knees were selected for detailed kinematic modeling. Subject-specific femoral and patellar point clouds were reconstructed from time-resolved CT data acquired during voluntary knee flexion. Within a predefined 5–15 mm neighborhood of the Schöttle point, a constrained sequential quadratic programming (SQP) approach was applied to identify an individualized femoral point (I-point) that minimized MPFL length variability while enforcing a femoral-surface constraint. **Results:** Compared with the Schöttle point, the I-point demonstrated a distinct spatial distribution, characterized primarily by a proximal shift along the femoral axis (PERMANOVA pseudo-F = 4.457, *p* = 0.006). Across 0–90° of knee flexion, the I-point was associated with reduced MPFL length variation and approached a relatively stable length-change profile near mid-flexion. **Conclusions:** These findings indicate that integrating 4D-CT-derived kinematics with constrained optimization can provide quantitative, imaging-based, motion-informed guidance for patient-specific femoral localization. This imaging-based framework may serve as a preoperative decision-support tool for personalized MPFLR planning.

## 1. Introduction

Patellar dislocation is most prevalent in adolescents and young adults, and nonoperative management is frequently associated with recurrent instability, with reported recurrence rates ranging from 15% to 69% [1]. The medial patellofemoral ligament (MPFL) is a primary soft-tissue stabilizer of the patella, contributing substantially to restraint against lateral displacement (up to 53–60% of the lateral dislocating force) [2]. MPFL injury has been reported in up to 87% of patients following a first-time dislocation, and approximately 67% of conservatively treated patients may experience recurrent dislocations that compromise function and quality of life [3]. Consequently, MPFL reconstruction (MPFLR) has become a mainstay surgical strategy to restore patellar stability and reduce recurrence [4,5].

Despite the widespread adoption of MPFLR, considerable heterogeneity remains in surgical techniques, and a universally accepted “gold standard” has not been established [6]. A major source of variability lies in femoral tunnel localization, which directly influences graft length-change patterns and mechanical behavior during knee motion [7]. The Schöttle point is the most commonly used radiographic landmark for femoral tunnel placement [8]; however, it is derived from static two-dimensional radiographs and exhibits non-negligible geometric variability [9]. Such variability may contribute to clinically relevant problems, including positioning-related failures [10] and nonphysiological isometry patterns that induce abnormal length–tension relationships [11]. More fundamentally, static radiographic landmarks cannot fully represent subject-specific trochlear morphology and in vivo patellar tracking, both of which are inherently dynamic and may vary substantially across individuals. These considerations motivate the development of motion-informed and individualized femoral localization strategies.

Recent advances in medical imaging and computational modeling provide an opportunity to address this gap. Data-driven approaches, including AI-based frameworks, have shown potential to enhance patient-specific modeling and decision support in clinical workflows [12,13]. In parallel, four-dimensional computed tomography (4D-CT) enables acquisition of time-resolved volumetric data and can capture joint motion trajectories across flexion–extension, offering access to in vivo kinematics that are not available from conventional static imaging [14]. For MPFLR planning, the key methodological question is not merely to propose an alternative fixed landmark, but to determine a femoral attachment that is both anatomically feasible and biomechanically favorable for a given patient’s patellofemoral motion [15].

In this study, we aimed to identify a motion-informed, patient-specific femoral fixation point selection in MPFLR by integrating 4D-CT-derived patellofemoral kinematics with constrained nonlinear optimization. Using 1382 healthy knee datasets, we constructed subject-specific 3D patellofemoral motion models and, in a representative subset of 58 knees, applied sequential quadratic programming (SQP) to identify an individualized femoral fixation point (I-point) that minimizes MPFL length variability while satisfying anatomical surface constraints. We then compared the resulting I-point with the conventional Schöttle point (S-point) in terms of spatial distribution and isometric stability across the flexion range. By providing quantitative evidence for motion-informed, patient-specific femoral localization, this work seeks to support more precise femoral tunnel placement and to facilitate future integration of computational assistance into personalized MPFLR planning.

## 2. Materials and Methods

### 2.1. Study Population

Healthy contralateral knees of patients with cruciate ligament ruptures were selected (not the general healthy population). Inclusion criteria: (1) age 18–50 years; (2) no prior knee surgery/symptoms; (3) normal patellar tracking on clinical exam. Exclusion criteria: (1) meniscal/ligamentous injuries; (2) severe traumatic/degenerative changes; (3) anatomical risk factors for MPFLR failure [5,16]. Demographic and anatomical characteristics were measured and analyzed descriptively (Table 1). This study was approved by the Institutional Review Board of The First Affiliated Hospital of Chongqing Medical University (Approval No. 2021-105) and was conducted in accordance with the Declaration of Helsinki (1964) and its subsequent amendments. The study was registered with the Chinese Clinical Trial Registry (ChiCTR2200063223).

### 2.2. 4D-CT Data Acquisition

Dynamic scanning was performed using a 320-detector row CT scanner (Aquilion ONE, Canon Medical Systems, Otawara, Japan). Participants were supine with fixed thighs and free-moving calves to complete natural knee motion cycles. Scanning parameters: slice thickness 0.5 mm, rotation time 0.35 s, 100 kV, 70 mA. Lead shielding was applied from the neck to the proximal thigh to minimize ionizing radiation exposure. Dynamic image acquisition and preprocessing were performed by a single radiologist to ensure consistency of imaging parameters. Each knee acquired 20–30 complete CT models per scan.

### 2.3. Establishment of 3D Patellofemoral Models and Landmark Identification

All CT datasets were exported in DICOM format and imported into Mimics (v26.0, Materialise, Leuven, Belgium). Bone segmentation was initially performed using threshold-based semi-automatic segmentation (Hounsfield unit threshold optimized for cortical bone), followed by manual refinement to correct boundary irregularities and remove artifacts. Segmentation was performed frame-by-frame to preserve dynamic geometric consistency.

Three-dimensional surface meshes were generated using non-destructive region-growing and hole-filling algorithms. Surface smoothing and mesh optimization were performed in Geomagic (3D Systems, Rock Hill, SC, USA) to reduce reconstruction noise while preserving anatomical contours.

The Schöttle point (S-point) was identified according to the original radiographic definition using anatomical references including the posterior cortical line extension, Blumensaat’s line, and the intersection of defined geometric constraints. Landmark identification was independently performed by three experienced orthopedic surgeons, and the mean coordinate value was used for analysis.

The patellar central reference point (C-point) was defined as the geometric centroid of the patellar articular surface. Its spatial trajectory across flexion was extracted for motion modeling.

### 2.4. Optimal Search for Femoral Attachment Points of the MPFL Based on SQP

To determine the optimal femoral attachment, I-point for MPFLR, a femur-centered local coordinate system was established with the origin at the midpoint of the medial (MFC) and lateral femoral condyles (LFC), and the XOY plane defined by the MFC, LFC, and reconstructed axis (RA); the patellar central reference point (C-point) was identified, and its spatial trajectory during knee motion was fitted with a function. The optimization objective was to minimize the variation in MPFL length using the standard deviation of these distances as the target function, where *C_i_* denotes the coordinates of the C-point in the i-th frame and *N* is the total number of frames. Constraint conditions were applied to ensure the I-point resided on the femoral surface (approximated via Delaunay triangulation) and within a 5–15 mm radius point cloud centered on the conventional S-point. The SQP algorithm was employed to solve this nonlinear optimization problem: a Lagrange function (with *λ* as Lagrange multipliers) was defined, and quadratic programming subproblems were constructed at each iteration point; the I-point and Lagrange multipliers were updated iteratively until the convergence criterion for the objective function was satisfied.

(1) $$f\left(P_{I}\right)=\sqrt{\frac{1}{N}\sum_{i=1}^{N}{\left(||P_{I}-P_{Ci}||-\frac{1}{N}\sum_{i=1}^{N}\left||P_{I}-P_{Ci}|\right|\right)}^{2}},$$

(2) $$g(P_{I})=[g_{1}(x_{1},y_{1},z_{1}),\ldots ,g_{M}(x_{M},y_{M},z_{M})]\leq 0,$$

(3) $$L(P_{I},\lambda )=f\left(P_{I}\right)+\sum_{i=1}^{N}\lambda_{i}g_{i}(P_{I}),$$

(4) $$\begin{array}{l} \min_{\Delta P_{I}}\frac{1}{2}\Delta P_{I}^{T}H^{k}\Delta P_{I}+(\nabla f(P_{I}^{k}))+\sum_{i=1}^{N}\lambda_{i}^{k}\nabla g_{i}(P_{I}^{k})\\ s.t.\;\nabla g_{i}{\left(P_{I}^{k}\right)}^{T}\Delta P_{I}+g_{i}(P_{I}^{k})\leq 0,i=1,\ldots ,N, \end{array}$$

### 2.5. Sample Size Calculation and Data Analysis

Sample size calculated via G*Power (v3.1.9.2): paired test (α = 0.05, power = 0.80, Cohen’s d = 0.5) requiring 34 models; expanded to 58 for robustness. Shapiro–Wilk tests confirmed non-normal coordinate distributions (S-point/I-point). PERMANOVA (primary) and pairwise Wilcoxon tests (preliminary) were used (*p* < 0.05). Distributions were visualized via MDS-KDE. Coordinates: X (medial–lateral), Y (anterior–posterior), Z (proximal–distal). Measurements: mm (rounded to 0.05). Software: R v4.3.2, Python v3.13.0.

## 3. Results

To address the primary aim, we evaluated whether a motion-informed optimization framework identifies a patient-specific femoral localization point associated with reduced MPFL length variability compared with the conventional Schöttle point.

### 3.1. Cohort Characteristics and Baseline Patellofemoral Morphology

Baseline demographic and morphological characteristics of the analyzed cohort (*n* = 58 knees) are summarized in Table 1. Participants had a mean age of 30.53 ± 7.34 years, including 39 males (67.24%) and 19 females (32.76%), with an equal distribution of left and right knees.

Patellar morphology according to Gelsamer and Wiberg classifications fell within reported physiological distributions. Quantitative indices—including patellar dimensions, epicondylar distance, Insall–Salvati index, patella trochlear index, trochlear groove angle, bisecting-offset index, patellar tilt, and tibial tuberosity–trochlear groove distance—were within established reference ranges (Table 1).

No knees demonstrated radiographic features consistent with patellofemoral malalignment. These findings indicate that the analyzed knees represent morphologically normal patellofemoral joints under the applied inclusion criteria.

### 3.2. 4D-CT-Based Motion Modeling and Point Identification

**Figure 1:**
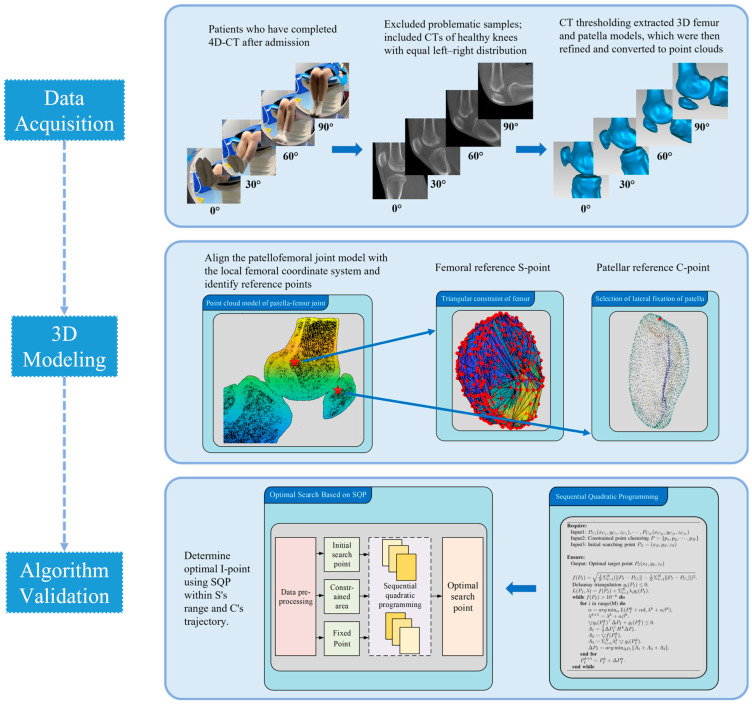
Experimental and computational procedures for 3D knee joint modeling. A total of 1382 knee CT models were studied for computation and analysis. 4D-CT was used to capture the patient’s active knee flexion from approximately 0° to 90°, and repair refinement was performed from the identified rough fuzzy 3D model. 3D models were converted to point clouds, identifying C-point (patella) and S-point (femoral reference). The individualized femoral I-point was calculated using a constrained SQP-based optimization method.

From 1382 available 4D-CT datasets, 58 knees were randomly selected for detailed modeling. Each knee included 20–30 consecutive frames acquired during active flexion–extension (Figure 1).

For each frame, a subject-specific 3D surface model of the patellofemoral joint was reconstructed and converted to a point cloud representation. The conventional femoral reference (Schöttle point; S-point) and a patellar central reference (C-point) were identified to calculate frame-wise MPFL length across 0–90° flexion. An individualized femoral fixation candidate (I-point) was then estimated using constrained nonlinear optimization within a predefined 5–15 mm neighborhood of the S-point, subject to a femoral surface constraint.

**Figure 2:**
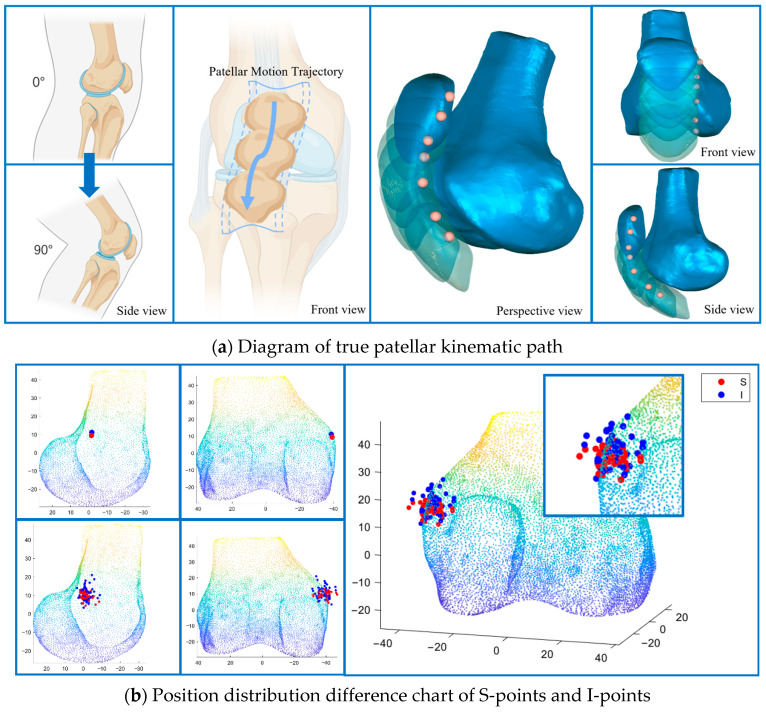
Patellar kinematics and femoral reference point distribution. (**a**) Schematic of in vivo patellar tracking during 0–90° knee flexion (3D reconstruction from 4D-CT). (**b**) Spatial distribution of Schöttle points (S-point, red) and individualized femoral points (I-point, blue) on a standardized femur (multiple projections); inset shows magnified local comparison.

As illustrated in Figure 2, S-points appeared relatively clustered, whereas I-points demonstrated broader spatial distribution across subjects. Formal statistical comparisons are presented below.

### 3.3. Spatial Distribution of Femoral Localization Points

PERMANOVA demonstrated a statistically significant difference between S-point and I-point coordinate distributions (pseudo-F = 4.457, *p* = 0.006).

Variance partitioning indicated that the observed separation was primarily attributable to the proximal–distal (*Z*-axis) component (95.1%), with substantially smaller contributions from the *X*-axis (4.0%) and *Y*-axis (1.0%).

Univariate analysis (Table 2) confirmed a significant difference in the Z-coordinate (I-point: 11.20 mm vs. S-point: 9.32 mm, *p* = 0.0027), consistent with a proximal shift along the proximal–distal axis. No significant differences were observed along the medial–lateral (X) or anterior–posterior (Y) axes (both *p* > 0.05).

**Figure 3:**
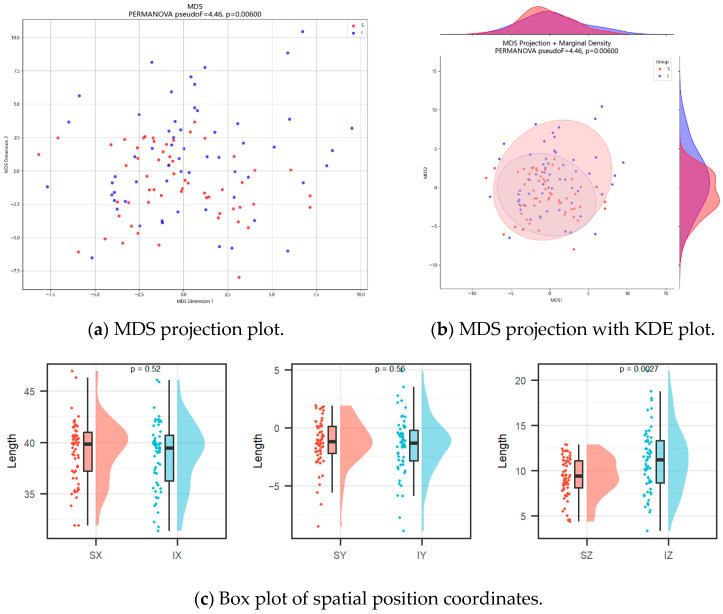
Coordinate position analysis of S-points and I-points. (**a**) Multidimensional scaling (MDS) projection of S-points and I-points. (**b**) Multidimensional scaling (MDS) projection with marginal kernel density estimate (KDE) plot of S-points and I-points. (**c**) Box plot of S-points and I-points *X*-axis, *Y*-axis and *Z*-axis coordinates.

Multidimensional scaling visualization with kernel density estimation is shown in Figure 3. The I-points demonstrated greater dispersion relative to S-points. As noted above, this distribution difference was predominantly driven by the *Z*-axis coordinate.

### 3.4. MPFL Length Variability Across Flexion

**Figure 4:**
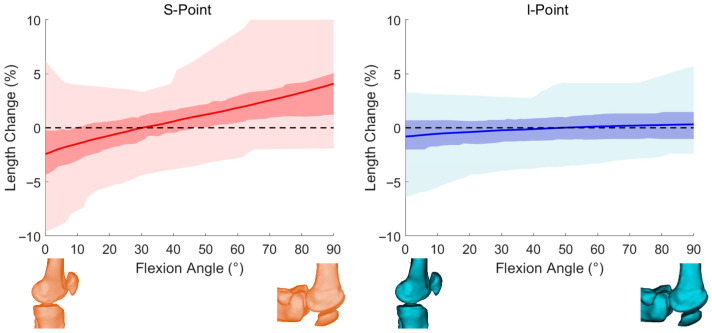
MPFL length variations linked by S-point and I-point. Length variation in MPFL linked by S-point and I-point during normal knee joint activity for all fitted samples.

MPFL length variation was normalized to the reference length (MPFL%) for both femoral attachment strategies. Across 0–90° of flexion, the S-point exhibited larger fluctuations in MPFL% than the I-point (Figure 4). At the subject level, the maximum MPFL% variation ranged from −9.41% to 9.14% for the S-point and from −6.44% to 5.68% for the I-point.

Angle-specific summaries are provided in Table 3. Briefly, the S-point showed greater shortening near full extension and progressively increased elongation toward deeper flexion, whereas the I-point remained closer to minimal length change across the motion range. Paired Wilcoxon signed-rank tests demonstrated statistically significant differences between the two strategies at each evaluated flexion angle (Table 3), with the largest separation observed at 0° and the I-point approaching minimal variation near mid-flexion (~50°) under the defined model.

These results indicate that, under the predefined optimization objective, the SQP-derived I-point was associated with reduced MPFL length variability compared with the conventional S-point. This finding reflects confirmation of the defined mathematical objective rather than independent biomechanical validation.

## 4. Discussion

This study investigated whether motion-informed, constrained optimization based on 4D-CT kinematics can refine femoral localization for medial patellofemoral ligament reconstruction beyond the conventional Schöttle point. Two principal observations emerged. First, the SQP-derived individualized femoral fixation point (I-point) demonstrated a spatial distribution that differed from the Schöttle point (S-point), with the between-point separation driven predominantly by the proximal–distal (*Z*-axis) component. Second, consistent with the predefined optimization objective, the I-point was associated with reduced modeled MPFL length variability across 0–90° knee flexion compared with the S-point.

Importantly, the reduction in MPFL length variability represents a confirmation of the constrained optimization framework under the defined objective function rather than an independent validation of improved biomechanical performance or surgical outcome. Therefore, the present study focuses on imaging-derived, motion-informed computational characteristics of femoral localization rather than clinical effectiveness.

Femoral tunnel placement substantially influences graft length behavior throughout functional knee motion and has been associated with postoperative stability and patient-reported outcomes in prior reports [17,18,19]. Although the Schöttle point remains the most widely used radiographic guideline [20,21,22], it is derived from static two-dimensional imaging and is subject to geometric variability [23,24]. Static landmarks also cannot capture inter-individual differences in patellar tracking during active motion, which arise from subject-specific joint morphology and neuromuscular control.

Rather than proposing another universal landmark, the current work presents a computational framework integrating time-resolved 4D-CT motion with constrained nonlinear optimization to identify a subject-specific femoral attachment within a clinically interpretable 5–15 mm neighborhood around the S-point. By enforcing a femoral-surface constraint, the I-point represents an optimized refinement of an established clinical reference rather than an unconstrained relocation, which may facilitate interpretability in computational planning workflows that currently rely on the Schöttle point as an initial estimate.

The observed spatial separation between I-points and S-points was supported by multivariate testing (PERMANOVA) and univariate coordinate comparisons. However, the difference was primarily attributable to the proximal–distal coordinates, while medial–lateral and anterior–posterior positions did not differ significantly. Thus, the spatial distinction should be interpreted as a predominantly unidirectional adjustment rather than a fully multidimensional redistribution.

The mean proximal shift (~2 mm) should be considered in the context of reported intraoperative femoral tunnel placement variability (approximately 3–5 mm). Whether such computational refinement exceeds routine surgical variability or translates into clinically meaningful differences remains uncertain and requires direct validation.

Across flexion, the I-point showed reduced modeled MPFL length fluctuation relative to the S-point under the defined computational conditions. Because nonphysiological graft length-change patterns have been associated with overconstraint or slackening at specific flexion angles [25,26,27,28], reduced variability may theoretically correspond to a more stable mechanical profile [29,30]. However, MPFL length in this study was estimated as a straight-line distance and did not account for ligament wrapping, soft-tissue interaction, graft routing, or physiological loading conditions. Therefore, the observed reduction should be interpreted as a modeled surrogate rather than evidence of improved in vivo graft behavior.

The broader dispersion of I-points across subjects reflects variability under subject-specific kinematic input within the optimization framework. Increased dispersion alone should not be interpreted as proof of improved accuracy or biomechanical superiority, and further investigation is needed to assess robustness against segmentation variability, measurement noise, and landmark identification uncertainty.

Several limitations merit consideration. Formal intra-observer and inter-observer reproducibility analyses were not performed, and residual measurement variability may influence optimization outputs. Knee motion during 4D-CT acquisition was voluntary, supine, and non-weight-bearing, which may differ from physiological loading conditions. Furthermore, no cadaveric, postoperative, or clinical outcome validation was conducted. Accordingly, the proposed framework should be considered preclinical and exploratory, and its clinical relevance requires prospective validation.

Future investigations should extend this framework to patellar instability cohorts, incorporate more realistic graft path modeling, assess robustness to segmentation variability, and determine whether computationally reduced MPFL length variability correlates with improved surgical precision or clinical outcomes in prospective studies.

In summary, integrating 4D-CT kinematics with constrained SQP optimization identifies a femoral fixation candidate point that differs systematically from the Schöttle point and demonstrates reduced modeled MPFL length variability under the defined computational conditions. While these findings provide quantitative insight into motion-informed femoral localization, their clinical impact remains to be established.

## Figures and Tables

**Table 1:** Statistical table of patient basic information and data table of various patella–tibial angles ^a^.

	Values ^b^
Age, y	30.53 [7.34]
Height, cm	169.33 [8.22]
Weight, kg	67.83 [13.65]
Sex	
Female	19 (32.76)
Male	39 (67.24)
Left and Right of Knee Joint Models	
Left	29 (50.00)
Right	29 (50.00)
Basic Data on the Patella and Femur	
Grelsamer Classification of the Patella	
I	39 (67.24)
II	10 (17.24)
III	9 (15.52)
Wiberg Classification of the Patella	
I	12 (20.69)
II	25 (43.10)
III	21 (36.21)
Patella Oblique Diameter, mm	40.58 [4.06]
Patella Transverse Diameter, mm	45.20 [3.05]
Intercondylar Distance Between the Medial and Lateral Epicondyles of the Femur, mm	81.11 [5.99]
Patella Height	
Insall–Salvati Index	0.94 [0.09]
Patella Trochlear Index	0.70 [0.22]
Trochlear Groove Morphology	
Trochlear Groove Angle, Degrees	125.45 [7.92]
Axial Linear Displacement of the Patella	
Patella Bisecting-Offset (BO) Index	62.63 [10.62]
Axial Tilt of the Patella	
Patella Tilt Angle, Degrees	9.81 [4.71]
Lateralization of the Tibial Tuberosity	
Tibial Tuberosity–Trochlear Groove Distance, mm	9.74 [6.48]

^a^ The normal ranges for the following parameters in healthy knees are provided: Insall–Salvati Index (0.8–1.2), Patella Trochlear Index (0.125–0.80), Trochlear Groove Angle (138° ± 6°), Patella Bisecting-Offset (BO) Index (44–66), Patella Tilt Angle (<12°), and tibial tuberosity–trochlear groove distance (<20 mm). The data indicate that all included patients had normal knees, with an equal distribution of left and right knees, and no knees exhibited pathological conditions. ^b^ Values are presented as mean [std. deviation] and number (percentage).

**Table 2:** Statistical table of coordinate position analysis of S-points and I-points.

	Femoral Reference Points					
	S (S_X_, S_Y_, S_Z_)		I (I_X_, I_Y_, I_Z_)		P ^c^	PERMANOVA ^d^
	Mean (95% CI) ^a^	Cov ^b^	Mean (95% CI)	Cov		
X	39.15 (38.36, 39.94)	1.5727	38.77 (37.91, 39.63)	1.5376	0.52	Pseudo-F = 4.457 P = 0.006
Y	−1.29 (−1.83, −0.74)	0.0045	−1.48 (−2.14, −0.82)	0.0064	0.56	
Z	9.32 (8.74, 9.89)	0.0041	11.20 (10.24, 12.15)	0.0134	0.0027	

^a^ 95% CI represents the 95% confidence interval of the mean. ^b^ Cov denotes covariance, reflecting differences between coordinate positions. ^c^ P denotes the probability value from the paired statistical test. ^d^ Pseudo-F denotes the PERMANOVA test statistic, and P represents the permutational *p*-value derived from 999 Monte Carlo permutations.

**Table 3:** Statistical table of coordinate dispersion and MPFL length change in S-points and I-points during normal knee joint activity.

Angle	Femoral Reference Points		
	S (54.84 ± 4.46 mm)	I (55.05 ± 4.34 mm)	P ^b^
	MPFL% ^a^	MPFL%	
0°	−2.33	−0.80	1.92 × 10^−9^
10°	−1.47	−0.56	2.22 × 10^−8^
20°	−0.71	−0.39	5.01 × 10^−3^
30°	−0.01	−0.23	4.37 × 10^−3^
40°	0.58	−0.12	7.23 × 10^−6^
50°	1.13	0.02	2.09 × 10^−7^
60°	1.60	0.12	3.17 × 10^−8^
70°	1.97	0.20	1.70 × 10^−8^
80°	2.23	0.26	8.59 × 10^−9^
90°	2.41	0.32	6.83 × 10^−9^

^a^ Mean MPFL percentage per 10° angular interval. ^b^ Paired Wilcoxon signed-rank test comparing ΔL% between S-point and I-point within subjects at matched flexion angles.

## Data Availability

The derived data supporting the findings of this study, including femoral and patellar coordinate points and MPFL length measurements, are provided in the Supplementary Materials. The original 4D-CT imaging data are not publicly available due to ethical and privacy restrictions.

## Supplementary Materials

The following supporting information can be downloaded at https://www.mdpi.com/article/10.3390/diagnostics16040508/s1: Figure S1: Length variation of the MPFL linked to the S-point and I-point during normal knee flexion in representative individual samples; Table S1: Detailed numerical data of MPFL length variation linked to the S-point and I-point during normal knee flexion.

## Abbreviations

The following abbreviations are used in this manuscript:

4D-CT	four-dimensional computed tomography;
MPFL	medial patellofemoral ligament;
MPFLR	medial patellofemoral ligament reconstruction;
S-point	Schöttle point;
I-point	individualized femoral point;
C-point	patellar central reference point;
SQP	sequential quadratic programming;
PERMANOVA	permutational multivariate analysis of variance;
MDS	multidimensional scaling;
KDE	kernel density estimation;
CI	confidence interval.
