# Supplementary material for: Motion-Informed, Patient-Specific Femoral Localization for MPFL Reconstruction Using 4D-CT and Constrained Optimization
Source: Diagnostics (Basel). 2026 Feb 7;16(4):508. doi: 10.3390/diagnostics16040508 (PMC12939497; doi:10.3390/diagnostics16040508)
Supplement: Supplementary file 1 [file diagnostics-16-00508-s001.zip › diagnostics-4129381-supplementary/supplementary/supplement Figure S1.pdf]

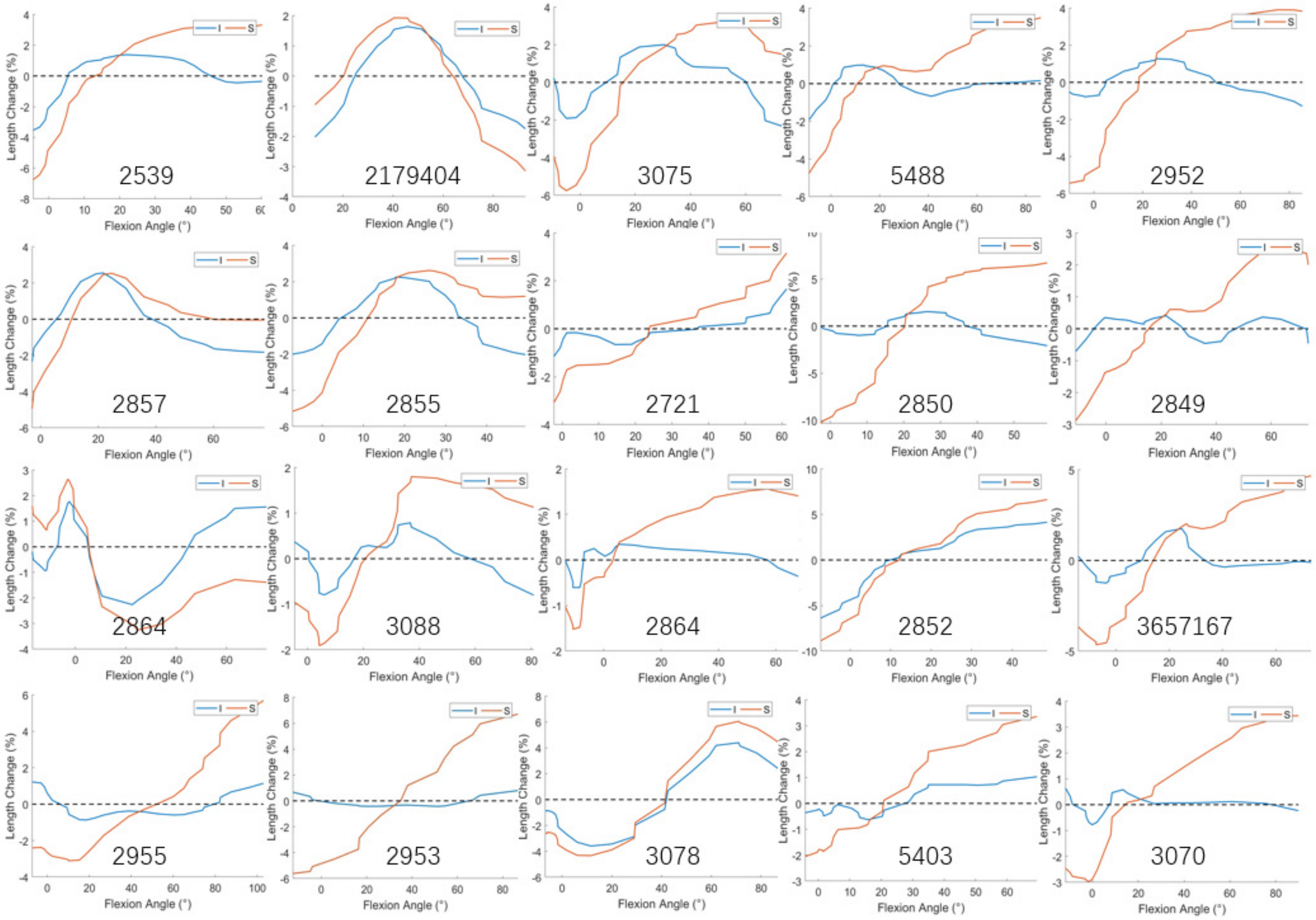

Supplement Figure S1. Length Variation of MPFL Linked by S-point and I-point During Normal Knee Joint Activity for some Single Samples
